# Decoding the Clinical and Therapeutic Significance of MEAK7 in Triple-Negative Breast Cancer Through Integrative Bioinformatics

**DOI:** 10.3390/biology15070543

**Published:** 2026-03-28

**Authors:** Durmus Ayan, Meltem Uyaner Kan, Ergul Bayram, Sibel Soylemez

**Affiliations:** 1 Medical Biochemistry, Faculty of Medicine, Nigde Omer Halisdemir University, Nigde 51200, Türkiye; durmusayan@hotmail.com; 2 Medical Biochemistry, Nigde Omer Halisdemir University Research and Training Hospital, Nigde 51200, Türkiye; eyaylagul@hotmail.com; 3 Gazi University Life Sciences Application and Research Center, Ankara 06560, Türkiye; soylemezsibel@gmail.com; 4 Medical Laboratory Techniques Program, Department of Medical Services and Techniques, Gazi University Vocational School of Health Services, Ankara 06560, Türkiye

**Keywords:** MEAK7, breast cancer, breast cancer subtypes, breast cancer therapy, triple negative breast cancer

## Abstract

Triple-negative breast cancer (TNBC) represents a clinically challenging breast cancer (BC) subtype, characterized by aggressive behavior, high recurrence risk, and limited therapeutic options. MEAK7 has been identified as an alternative mTORC1 signaling pathway regulator; however, its role in BC and TNBC remains uninvestigated. This study aims to assess MEAK7 expression, prognostic significance, and therapeutic potential. We employed public datasets, including TCGA, bc-GenExMiner v5.2, GEPIA3, DOSurvive platforms, Kaplan–Meier Plotter, UALCAN, TIMER2.0, STRING, ENCORI, HPA, miRDB, TargetScan, and CRISPRdb. MEAK7 expression was significantly elevated in BC tissues versus normal breast tissue. MEAK7 expression was pronounced in TNBC and basal-like subtypes, with hypomethylation of its promoter region in TNBC. Elevated MEAK7 expression correlated with reduced disease-free survival (DFS) in TNBC and basal-like. Multivariate Cox regression identified MEAK7 as a significant prognostic factor for overall survival, independent of age and tumor stage. MEAK7 showed CRISPR-targetable gRNA profiles with high on-target efficiency and minimal off-target effects. Analyses revealed negative correlation with tumor-suppressive RNAs (miR-149-3p, miR-135a-5p, and LINC00993) and positive correlation with aggressive regulators (miR-135b-5p and HIF1A-AS2). This study represents one of the initial comprehensive and multi-platform bioinformatic analyses demonstrating that MEAK7 exhibits elevated expression in breast cancer, particularly within the aggressive TNBC. The findings indicate that MEAK7 may serve as a promising prognostic biomarker in TNBC biology and suggest its viability as a molecular candidate for future investigation in targeted therapeutic strategies.

## Simple Summary

Triple-negative breast cancer is a type of breast cancer that tends to be more aggressive and difficult to treat because it lacks the most commonly targeted hormone receptors. Patients face a higher risk of early recurrence and treatment options are limited. In this study, we investigated the potential role of a gene called MEAK7 (mTORC1-associated protein; EAK-7 homolog, HGNC-approved gene symbol; also known as KIAA1609 and TLDC1) in this aggressive type of cancer. By analyzing comprehensive, publicly available cancer databases, we determined that MEAK7 levels are higher in breast cancer tissues compared to normal breast tissue. This increase was more pronounced for triple-negative and basal-like subtypes. It was observed that patients with high MEAK7 levels had shorter disease-free survival. We also obtained molecular findings suggesting that MEAK7 may be linked to other genetic regulators associated with tumor growth and poor clinical outcomes. Computer-based analyses also suggest that MEAK7 could be targeted in the future using modern gene editing approaches. Although these results require validation through laboratory and clinical studies, our findings indicate that MEAK7 may be a promising candidate for predicting disease course and supporting the development of new targeted therapies in aggressive breast cancer.

## 1. Introduction

Breast cancer (BC) is a complex disease that presents significant clinical challenges in terms of diagnosis, treatment, and prognosis due to its molecular heterogeneity [1]. In particular, triple-negative breast cancer (TNBC) is characterized by the absence of estrogen receptor (ER), progesterone receptor (PR), and HER2 expression, and is associated with aggressive biological behavior, high metastatic potential, and limited treatment options [2,3,4]. In the TNBC, where targeted therapies are largely ineffective, the identification of new molecular biomarkers and therapeutic targets stands out as a critical need [5,6,7].

The dysregulated activation of signaling pathways associated with cell proliferation, metabolic reprogramming, and stress adaptation plays a fundamental role in the pathogenesis of TNBC [8,9,10]. In this context, the PI3K/AKT/mTOR axis is one of the most frequently activated oncogenic pathways in TNBC and is strongly associated with tumor growth, treatment resistance, and poor clinical outcomes [11,12]. In particular, the increase in mTORC1 activity contributes to the acquisition of an aggressive phenotype by TNBC cells through the support of protein synthesis and anabolic metabolism [13,14].

MEAK7 (mTORC1-associated protein; EAK-7 homolog, HGNC-approved gene symbol; also known as KIAA1609 and TLDC1) is a relatively new and limitedly studied protein, identified as an alternative and mTORC2-independent regulator of mTORC1 signaling [15]. Research indicates that MEAK7 may establish a functional association with mTOR signaling components, potentially forming an alternative mTOR complex that facilitates cellular growth and metabolic adaptation. This is evidenced by the activation of mTOR downstream effectors, such as S6K2 and 4E-BP1, in human cells [15,16]. Although the upregulated MEAK7 gene level has been reported to be associated with lymph node metastasis in BC [17], current knowledge regarding the expression profile and clinical significance of MEAK7 in solid tumors is quite limited; in particular, the biological and prognostic role of MEAK7 in the context of TNBC has not been systematically investigated.

In recent years, the use of high-throughput transcriptomic data and integrated bioinformatics analyses in cancer biology has provided a powerful approach for revealing the functional roles and clinical significance of novel genes [18,19,20]. However, in the literature, there is no comprehensive study evaluating the expression pattern of MEAK7 in BC in comparison to normal tissue, examining its distribution among molecular subtypes (especially TNBC), demonstrating its prognostic value through survival analysis, and thoroughly analyzing the associated signaling pathways.

In this study, the expression profile of MEAK7 in BC was analyzed in detail using large-scale transcriptomic data based on TCGA and GTEx. In addition, the relationship of MEAK7 expression with the TNBC, its impact on patient survival, co-expressed gene networks, and associated biological pathways were systematically evaluated through multilayered bioinformatic approaches. In addition to shedding light on the potential role of MEAK7 in TNBC biology, the findings are intended to provide an initial molecular basis for future experimental studies and targeted therapeutic strategies.

## 2. Materials and Methods

### 2.1. Data Acquisition and Gene Expression Analysis

The expression profile of the MEAK7 gene in BC was analyzed using RNA-seq data obtained from The Cancer Genome Atlas (TCGA-BRCA) and Genotype-Tissue Expression (GTEx) projects. Differential expression analysis between tumor and normal breast tissues was performed through GEPIA3 (https://gepia3.bioinfoliu.com/ accessed on 12 January 2026), a web-based platform. Default parameters were used in GEPIA3 analyses; |log2 fold change| ≥ 1 and *p* < 0.05 thresholds were set as the criteria for statistical significance [21]. Differential gene expression analysis was conducted utilizing the GEPIA3 web server, which employs the DESeq2 methodology. Statistical significance was evaluated using the Wald test, and *p*-values were adjusted for multiple comparisons using the Benjamini–Hochberg false discovery rate method. Expression values were reported as log2(TPM + 1).

The Human Protein Atlas (HPA) database was used to validate transcriptomic findings at the protein level [22]. MEAK7 protein expression was qualitatively evaluated based on immunohistochemical (IHC) staining images of normal breast tissue and breast cancer tissues. Using the antibody-based IHC data presented in the HPA, MEAK7 protein expression levels were classified as low, medium, and high, and their consistency with gene expression data was analyzed comparatively.

### 2.2. Clinicopathological Subgroup and Stages Analysis

The UALCAN platform (https://ualcan.path.uab.edu/ accessed on 12 January 2026) was used to evaluate the relationship between MEAK7 expression and clinical and pathological subgroups [23]. In this context, MEAK7 expressions were compared based on molecular subtypes (Luminal A, Luminal B, HER2-enriched, and TNBC), tumor stage and patient demographic characteristics. In all analyses, TCGA normalized transcriptomic data provided by the platform were used as the basis, and a *p* value of <0.05 was considered statistically significant. Clinicopathological subgroup analyses of MEAK7 expression were conducted utilizing the UALCAN web portal, which is based on TCGA normalized RNA-seq data. Differences between two groups were evaluated using Student’s *t*-test, while comparisons among multiple groups were performed using one-way ANOVA. A *p*-value of less than 0.05 was considered to indicate statistical significance.

### 2.3. DNA Methylation Analysis

The DNA methylation profile of the MEAK7 gene was analyzed using data from The Cancer Genome Atlas Breast Invasive Carcinoma (TCGA-BRCA) cohort. Comparative methylation analyses were performed using data containing β-values based on the Illumina HumanMethylation450 BeadChip platform, obtained from TCGA, through the UALCAN web-based tool (https://ualcan.path.uab.edu/ accessed on 12 January 2026) [23]. Methylation levels of the promoter region of the MEAK7 gene were evaluated comparatively between normal breast tissues and BC tissues. In addition, MEAK7 promoter methylation levels were analyzed based on molecular subtypes (Luminal, HER2-enriched, and TNBC) and pathological tumor stages. Methylation levels were expressed as β-values (ranging from 0 to 1), with higher β-values representing increased DNA methylation. In all analyses, the built-in statistical algorithms of the UALCAN platform were used, and a *p*-value of < 0.05 was considered statistically significant.

### 2.4. Survival Analysis

The Kaplan–Meier plotter (https://kmplot.com/analysis/ accessed on 12 January 2026) is proficient in evaluating the association between the expression of all genes (mRNA, miRNA, protein, and DNA) and survival across over 40,000 samples from 21 tumor types [24]. To evaluate the prognostic effect of MEAK7 expression levels, survival analyses were conducted. Patients were divided into high and low expression groups according to the median MEAK7 expression level. Survival analyses were performed utilizing the Kaplan–Meier Plotter database. Patients were categorized into high- and low-expression cohorts based on the median expression value of MEAK7. Kaplan–Meier survival curves were constructed, and intergroup differences were evaluated using the log-rank test. Hazard ratios (HRs) with 95% confidence intervals (CIs) were determined through a univariate Cox proportional hazards model. The JetSet best probe was employed for microarray-based analyses.

### 2.5. Gene Expression Analysis with the DOSurvive Database

To evaluate the relationship between gene expression and survival in TNBC, the DOSurvive (Disease Outcome & Survival Visualization) web-based database was used (https://dosurvive.lab.nycu.edu.tw/gene# accessed on 12 January 2026). This platform serves as a tool that visualizes the relationships between gene expression and clinical outcomes across TCGA, GEO, and other publicly available cancer genomic datasets. Survival analyses were conducted utilizing the DOSurvive web platform [25]. Patients were categorized into high- and low-expression cohorts based on the median expression value of MEAK7. Kaplan–Meier survival curves were generated, and intergroup differences were assessed using the log-rank test. Univariate and multivariate Cox proportional hazards regression analyses were performed to evaluate the independent prognostic significance of MEAK7, adjusting for age and tumor stage.

### 2.6. Targeted Expression Analysis

We utilized bc-GenExMiner v 5.2 web tool (https://bcgenex.ico.unicancer.fr/BC-GEM/GEM-Accueil.php?js=1 accessed on 12 January 2026) for analysis of targeted expression and gene correlation targeted. bc-GenExMiner v5.2 is a statistical analysis tool designed for examining annotated BC transcriptomic data, including DNA microarrays (*n* = 11,552) and RNA-seq datasets (*n* = 5023). It enables users to investigate the expression of specific genes of interest in the context of BC [26,27]. Gene expression and correlation analyses were conducted utilizing the bc-GenExMiner v5.2 platform, which incorporates extensive BC transcriptomic datasets obtained from DNA microarrays and RNA sequencing. Differences between two groups were evaluated using Welch’s *t*-test, while comparisons across multiple groups were executed using one-way ANOVA. Correlation analyses employed Pearson or Spearman correlation coefficients, as appropriate. A *p*-value of less than 0.05 was deemed statistically significant.

### 2.7. CRISPR gRNA Design and Targetability Analysis

To assess CRISPR-Cas9-mediated modulation of target genes, we used CRISPRdb (https://crisprdb.org/cgi-bin/search_gRNA.cgi; accessed on 12 January 2026) to identify guide RNA (gRNA) sequences targeting genes including MEAK7. CRISPRdb integrates genomic context and off-target prediction to select efficient gRNAs. Coding sequences from Ensembl database (GRCh38/hg38) were submitted to CRISPRdb with SpCas9 parameters. gRNAs were filtered based on NGG protospacer adjacent motif (PAM). For each gene, we selected top 20 gRNA candidates with highest on-target scores and lowest off-target burden. Off-target assessment used genome-wide searches with three mismatches outside seed region, excluding sequences with coding region hits. gRNAs were evaluated for GC content and secondary structure. Final selection used composite ranking of on-target score, off-target risk, and sequence features. This evaluation supports subsequent experimental validation and translational potential of CRISPR-based targeting in BC [28]. Furthermore, CRISPR-based guide RNA (gRNA) design was conducted to identify potential sequences targeting the MEAK7 gene. This analysis was included as a preliminary translational step to support potential future functional validation studies involving MEAK7.

### 2.8. Prediction of MEAK7 miRNA Interactions Using TargetScan 8.0 and miRDB

Predicted miRNA–mRNA interactions were investigated utilizing the TargetScanHuman database (Release 8.0; https://www.targetscan.org; accessed on 12 January 2026). TargetScan identifies potential binding sites in mammalian transcripts by detecting canonical 8mer, 7mer, and 6mer seed matches, prioritizing candidate miRNAs through context++ scoring. Release 8.0 uses an enhanced biochemical model integrating seed pairing, site accessibility, 3′-UTR features, and conservation for target ranking. Family level prediction files were downloaded, extracting the cumulative weighted context++ score (CWCS) and probability of conserved targeting (PCT) for genes of interest. Lower CWCS values indicate greater predicted inhibitory activity for miRNA regulation [29].

In addition to the TargetScan analysis, miRNA target predictions were obtained from miRDB (http://mirdb.org; accessed on 12 January 2026), a resource utilizing machine learning and trained on high-throughput CLIP-Seq experimental datasets. miRDB assigns a target score (50–100) indicating miRNA binding confidence based on its MirTarget model. For each gene, miRNAs scoring ≥80 were considered high-confidence regulators. Predictions were downloaded using default parameters, and miRNAs found by both TargetScan and miRDB were prioritized for analyses [30].

### 2.9. LncRNAs Associated with Triple-Negative Breast Cancer Identified Through the LncRNADisease Database

The LncRNADisease database (http://www.cuilab.cn/lncrnadisease; accessed on 12 January 2026) was employed to identify long non-coding RNAs associated with BC. This resource compiles experimentally validated associations between lncRNAs and human diseases and offers computational tools for predicting potential lncRNA–disease relationships. Additionally, the platform integrates multilayer interaction data for each lncRNA, encompassing its interactions with DNA, RNA, proteins, and miRNAs [31].

To investigate the lncRNA-mediated regulatory networks of the MEAK7 gene, the LncNet tool within the LncExpDB Version 2.0 database (https://ngdc.cncb.ac.cn/lncexpdb/; accessed on 12 January 2026) was used. LncNet (https://ngdc.cncb.ac.cn/lncexpdb/lncnet; https://ngdc.cncb.ac.cn/lncexpdb/; accessed on 12 January 2026) visualizes lncRNA–mRNA interaction networks for specified target genes using Spearman correlation coefficients to identify reliable co-expression patterns. MEAK7 was defined as the target gene for correlation analysis across database samples. LncRNAs with significant associations with MEAK7 were identified using correlation coefficients and statistical significance. The platform’s default thresholds were applied for network construction. The resulting lncRNA–MEAK7 co-expression network was analyzed to evaluate regulatory mechanisms and identify candidate regulatory axes, aiming to reveal MEAK7’s potential role in cancer biology [32].

### 2.10. ENCORI-Based Identification of CLIP-Validated miRNA–mRNA Interactions

The regulatory interactions between miRNA and mRNA for MEAK7 were investigated using the ENCORI database (also known as starBase) (https://starbase.sysu.edu.cn; accessed on 12 January 2026). ENCORI integrates CLIP-seq datasets (AGO-CLIP, PAR-CLIP, HITS-CLIP, iCLIP), RNA-RNA interaction data, and transcriptomic resources to identify miRNA binding sites. The “miRNA-mRNA” module retrieves target gene interactions with CLIP-seq evidence, filtered by platform settings requiring supporting CLIP experiments and ENCORI significance criteria [33]. miRNAs consistently detected across multiple CLIP datasets are considered high-confidence regulators. All data tables are exported in text format and subsequently cross-validated against computational predictions from TargetScan and miRDB.

## 3. Results

### 3.1. Differential Gene and Protein Expression of MEAK7 in Breast Cancer Tissues

**Figure 1:**
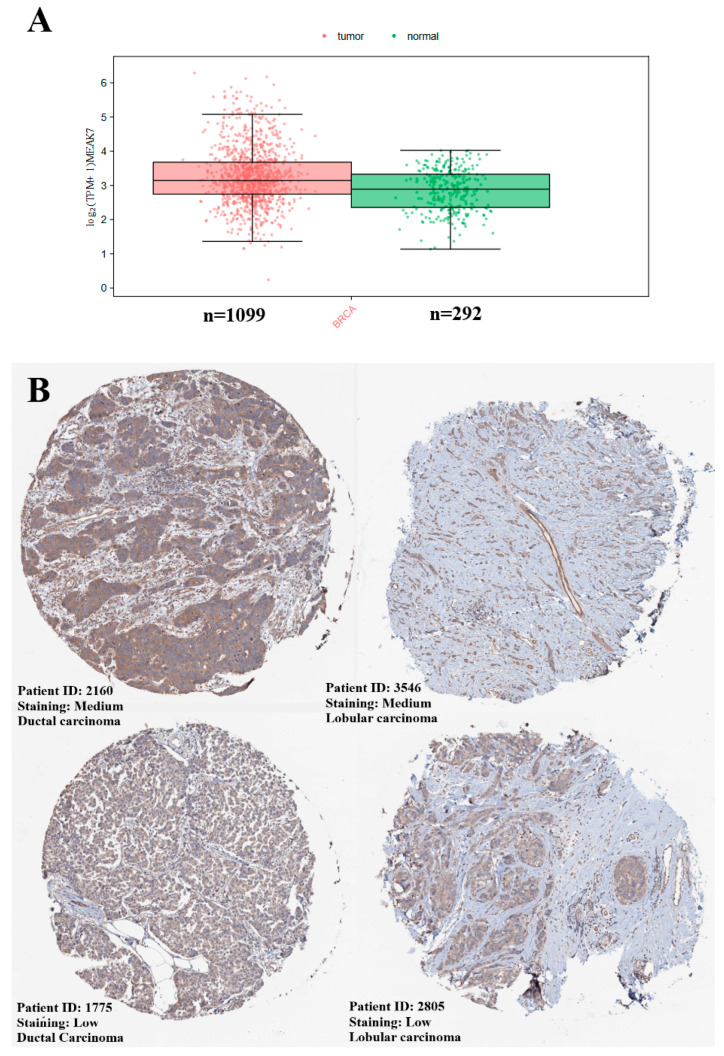
(**A**) Differential mRNA expression of MEAK7 between breast cancer tissues and normal breast tissues based on TCGA-BRCA and GTEx datasets, analyzed using the GEPIA3 platform. Expression levels are presented as log2-transformed transcripts per million (TPM). Red dots represent tumor samples, and green dots represent normal breast tissue samples. Sample sizes for each group are indicated below the box plots. (**B**) Representative immunohistochemical (IHC) staining images of MEAK7 protein expression in breast cancer tissues obtained from the Human Protein Atlas (HPA) database. Images include ductal carcinoma and lobular carcinoma samples with medium and low staining intensities. Patient IDs and staining intensity levels are provided as indicated BRCA (Scale bar = 200 μm).

The expression profile of MEAK7 in BC was comprehensively evaluated at both gene and protein levels. In analyses conducted at the transcriptomic level, MEAK7 gene expression was comparatively examined between tumors and normal adjacent breast tissues. These analyses were performed using RNA-seq data from TCGA-BRCA tumor samples and GTEx normal breast tissues via the GEPIA3 platform. Expression comparisons in GEPIA3 were interpreted based on median log_2_(TPM + 1) values derived from RNA-seq data. The results showed that MEAK7 expression levels were elevated in tumor tissue (median = 3.14) compared to normal tissue (median = 2.89; *p* = 2.66 × 10^−14^) (Figure 1A). To validate the transcriptomic findings at the protein level, MEAK7 protein expression was assessed using the HPA database. According to IHC data from ductal and lobular BC tissues, MEAK7 protein levels were classified under the low and medium expression categories (Figure 1B).

### 3.2. Association of MEAK7 Expression with Molecular Subtypes, Stages and Age of Breast Cancer

**Figure 2:**
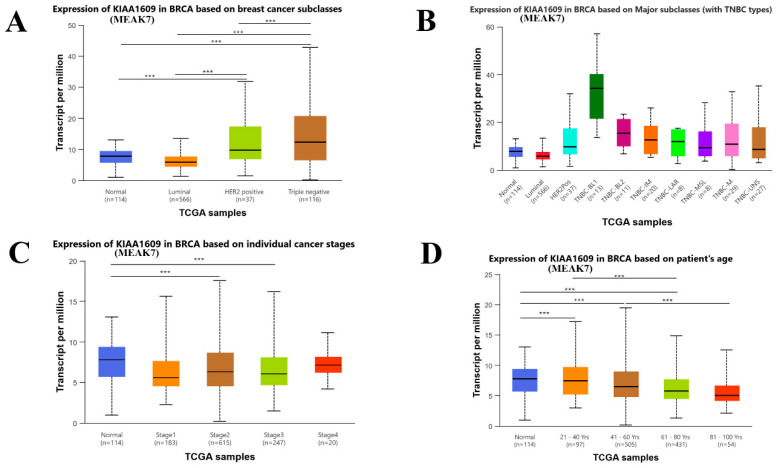
Expression patterns of MEAK7 in breast cancer based on molecular subtypes and tumor stages. (**A**) MEAK7 mRNA expression levels across normal breast tissue and major breast cancer molecular subtypes, including Luminal, HER2-positive, and triple-negative breast cancer (TNBC), based on TCGA-BRCA dataset. Expression values are presented as transcripts per million (TPM). (**B**) MEAK7 mRNA expression profiles across major breast cancer subclasses with detailed TNBCs, including TNBC-BL1, TNBC-BL2, TNBC-IM, TNBC-LAR, TNBC-MSL, TNBC-M, and TNBC-UNS, using TCGA-BRCA samples. Expression levels are shown as TPM. (**C**) MEAK7 mRNA expression levels in breast cancer according to individual pathological tumor stages (Stage I–IV) compared with normal breast tissue, based on TCGA-BRCA cohort. (**D**) MEAK7 mRNA expression levels in breast cancer according to patient age groups, including 21–40 years, 41–60 years, 61–80 years, and 81–100 years, compared with normal breast tissues, based on the TCGA-BRCA cohort. Expression levels are presented as transcripts per million (TPM). In all panels, box plots represent the median and interquartile range, with whiskers indicating the minimum and maximum values. Sample numbers for each group are indicated below the corresponding categories. Statistical significance was assessed using platform-specific statistical tests, and significance levels are denoted as *** for *p* < 0.05.

The relationship between MEAK7 expression and molecular subtypes of BC was analyzed using transcriptomic data from the TCGA-BRCA cohort. Subtype-based comparisons were performed via the UALCAN platform. Normal adjacent tissue as well as Luminal, HER2-positive, and TNBC were evaluated. The analysis results indicate that MEAK7 expression levels vary among the molecular subtypes. MEAK7 expression was notably elevated in TNBC (median = 12.3) compared to normal adjacent tissue (median = 7.8), luminal (median = 5.9), and HER2-positive (median = 9.7), with *p*-values of 1.86 × 10^−11^, 6.28 × 10^−12^, and 0.033, respectively. Furthermore, MEAK7 expression was decreased in normal adjacent tissue compared to the HER2-positive subtype (*p* = 0.00098). MEAK7 levels were significantly reduced in the Luminal compared to the HER2-positive (*p* = 0.00062) (Figure 2A).

Upon comparing MEAK7 levels across the primary subtypes of TNBC, it was observed that MEAK7 levels were statistically significantly reduced in normal adjacent tissue (median = 7.8) compared to the TNBC Basal-like 1, TNBC Basal-like 2, TNBC Immunomodulatory, TNBC Mesenchymal, and TNBC unspecified (median = 34.1, *p* = 0.0000027; median = 15.5, *p* = 0.018; median = 12.6, *p* = 0.0081; median = 10.8, *p* = 0.0019, and median = 8.7, *p* = 0.023, respectively). MEAK7 levels were significantly reduced in the luminal group (median = 5.9) compared to the TNBC Basal-like 1, TNBC Basal-like 2, TNBC Immunomodulatory, TNBC luminal androgen receptor, TNBC Mesenchymal, and TNBC unspecified (median = 34.1, *p* = 0.0000025; median = 15.5, *p* = 0.017; median = 12.6, *p* = 0.0070; median = 11.9, *p* = 0.026; median = 10.8, *p* = 0.0014, and median = 8.7, *p* = 0.017, respectively). When it comes to the differences among the major TNBCs themselves, MEAK7 expression levels were elevated in the TNBC Basal-like 1 subtype (median = 34.1) in comparison to the TNBC Basal-like 2, TNBC Immunomodulatory, and TNBC luminal androgen receptor subtypes, TNBC mesenchymal stem-like, TNBC Mesenchymal, and TNBC unspecified (median = 15.5, *p* = 0.015; median = 12.6, *p* = 0.0011; median = 11.9, *p* = 0.00011; median = 9.4, *p* = 0.00032; median = 19.2, *p* = 0.00000019; and median = 8.7, *p* = 0.000000093, respectively). There was no statistically significant change among the other subtypes (*p* > 0.05). In addition to TNBC, MEAK7 expression levels were significantly reduced in normal adjacent tissue (median = 7.8) compared to HER2-positive tissue (median = 9.7) (*p* = 0.00093). Furthermore, MEAK7 levels were significantly lower in HER2-positive tissue compared to TNBC-BL1 (*p* = 2.09 × 10^−9^) (Figure 2B).

According to the analysis based on the pathological subtypes of BC, MEAK7 levels were significantly elevated in the normal adjacent tissue (median = 7.8) compared to those in stage 2 and stage 3 tissues (median = 8.6, *p* = 0.0004 and median = 8.0, *p* = 0.0021, respectively). There was no statistically significant difference in MEAK7 levels among the other stages (*p* > 0.05) (Figure 2C).

When examining the relationship between age at BC diagnosis and MEAK7 levels, the MEAK7 levels were reduced in the tissues of patients diagnosed with BC at ages 21–40 (median = 7.4), 41–60 (median = 6.5), and 61–80 (median = 5.8) when compared to normal adjacent tissue (median = 7.8) (*p* = 0.012, *p* = 0.000064, and *p* = 0.0048, respectively). Conversely, MEAK7 levels were significantly elevated in the tissues of individuals diagnosed with BC at ages 21–40 compared to those aged 81–100 (median = 5.0) (*p* = 0.034). Similarly, MEAK7 levels were significantly elevated in the tissues of those diagnosed with BC at ages 41–60 compared to those aged 81–100 (*p* = 0.031) (Figure 2D).

### 3.3. Promoter DNA Methylation Levels of MEAK7 in Breast Cancer

**Figure 3:**
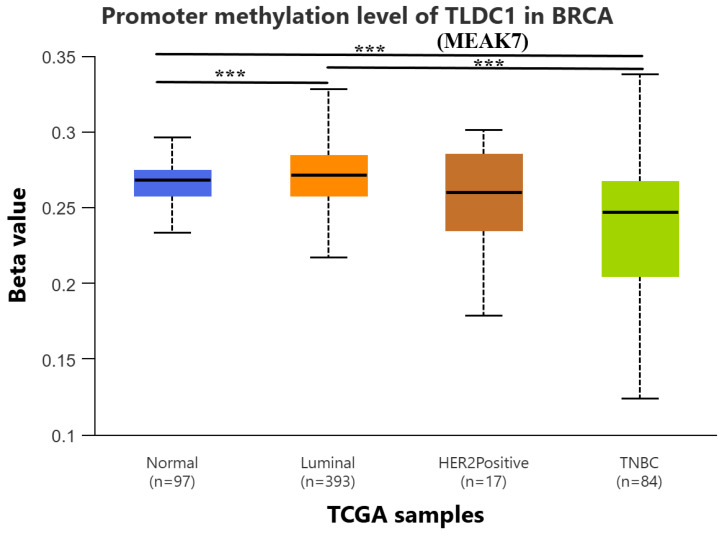
Promoter DNA methylation levels of the MEAK7 gene in normal breast tissues and breast cancer molecular subtypes, including Luminal, HER2-positive, and Triple-Negative Breast Cancer (TNBC), based on the TCGA-BRCA cohort. DNA methylation data were obtained from Illumina HumanMethylation450 BeadChip arrays and analyzed using the UALCAN platform. Methylation levels are presented as β-values, ranging from 0 (unmethylated) to 1 (fully methylated). Box plots represent the median and interquartile range, with whiskers indicating the minimum and maximum values. Sample sizes for each group are shown below the corresponding categories. Statistical significance between groups was assessed using Student’s *t*-test, and *p*-values < 0.05 were considered statistically significant. Significance levels are denoted as *** for *p* < 0.05.

The DNA methylation levels of the promoter region of the MEAK7 gene were comparatively analyzed between normal breast tissues and BC subtypes. The methylation levels of the MEAK7 promoter in normal adjacent tissue were significantly higher compared to those in TNBC (*p* = 0.0017). Furthermore, the MEAK7 promoter demonstrated elevated methylation levels in luminal subtypes relative to TNBC (*p* = 0.000096). Additionally, the methylation levels of the MEAK7 promoter were found to be significantly higher in normal adjacent tissue compared to luminal subtypes (*p* = 0.025). There were no statistically significant differences in the other subtype comparisons (*p* > 0.05) (Figure 3). Due to the small sample size of the HER2-positive subgroup (n = 17), these findings should be interpreted with caution and regarded as exploratory.

### 3.4. Prognostic Significance of MEAK7 Expression in Breast Cancer

**Figure 4:**
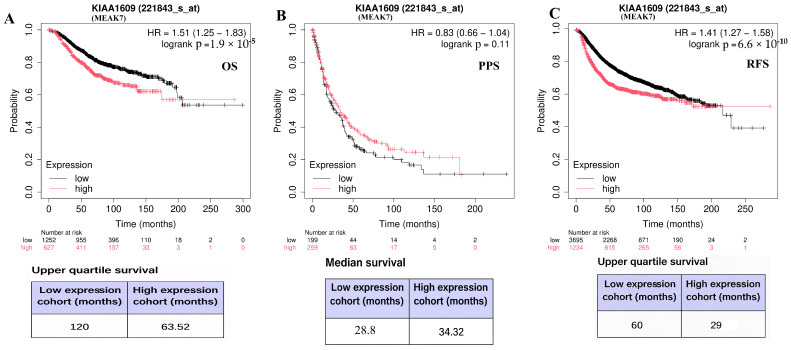
Prognostic significance of MEAK7 expression in breast cancer. (**A**) Overall survival (OS) analysis showing × 10^−5^ survival differences between low and high MEAK7 expression groups. (**B**) Post-progression survival (PPS) analysis according to MEAK7 expression levels. (**C**) Recurrence-free survival (RFS) analysis based on MEAK7 expression status. Hazard ratios (HRs) with 95% confidence intervals (CIs) and log-rank *p*-values are displayed within each panel. The number of patients at risk is shown below the Kaplan–Meier curves. Survival time is expressed in months. Tables below the curves indicate upper quartile or median survival times for the corresponding expression groups, as provided by the Kaplan–Meier Plotter platform.

Kaplan–Meier survival analyses assessed MEAK7 expression’s prognostic significance in breast cancer using the Kaplan–Meier Plotter database. The probe 221843_s_at (JetSet best probe) was used, with patients categorized into high- and low-expression groups based on median expression. Hazard ratios (HRs) with 95% confidence intervals (CIs) and log-rank *p*-values are shown. Patient numbers at risk are indicated below the curves, with survival time in months. Tables show upper quartile or median survival times for expression groups, as provided by the platform. The analysis revealed that elevated MEAK7 levels were statistically correlated with reduced overall survival (OS) [HR = 1.51 95% CI (1.25–1.83)] and relapse-free survival (RFS) [HR = 1.41 95% CI (1.27–1.58)] in BC (*p* = 0.000019 and *p* = 6.6 × 10^−10^, respectively). Conversely, MEAK7 expression levels did not exhibit a statistically significant association with post-progression survival (PPS) [HR = 0.83 95% CI (0.66–1.04), *p* = 0.11)] in BC Figure 4).

### 3.5. The Relationship of MEAK7 Expression with Survival, Age, and Stage (DOSurvive Analysis)

**Figure 5:**
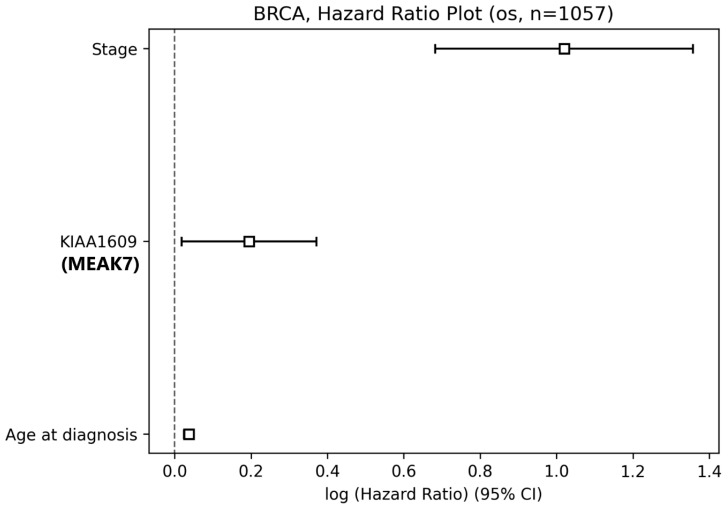
Multivariate Cox proportional hazards analysis of overall survival in breast cancer. Forest plot showing the results of multivariate Cox proportional hazards regression analysis for overall survival (OS) in breast cancer patients from the TCGA-BRCA cohort (*n* = 1057). The model included tumor stage, age at diagnosis, and MEAK7 expression as covariates. Squares represent hazard ratios (HRs), and horizontal lines indicate 95% confidence intervals (CIs). The vertical dashed line denotes HR = 1. The *x*-axis displays log-transformed hazard ratios. Statistical significance and corresponding HRs with 95% CIs were derived from the Cox regression model. ☐ Squares represent the estimated hazard ratios (HRs), while horizontal lines indicate the corresponding 95% confidence intervals.

The relationship between MEAK7 gene expression and survival outcomes in BC patients was evaluated using Kaplan–Meier analyses with the DOSurvive database. In these analyses, patients were divided into high and low expression groups according to MEAK7 expression levels. Multivariate Cox regression analysis demonstrated that MEAK7 expression remained an independent prognostic factor for overall survival after adjustment for tumor stage and age at diagnosis (HR = 1.21, 95% CI: 1.02–1.45, *p* = 0.031). Even in analyses adjusted for tumor stage and age, high expression of MEAK7 increases the risk of death by approximately 21%. Age at diagnosis was significantly associated with overall survival, with each one-year increase in age conferring a 4% higher risk of death (HR = 1.04, 95% CI: 1.02–1.05, *p* = 1.61 × 10^−8^). Tumor stage was strongly associated with overall survival, with advanced stage conferring a nearly 2.8-fold increased risk of death (HR = 2.77, 95% CI: 1.98–3.88, *p* = 3.18 × 10^−9^) (Figure 5).

### 3.6. Validation of Target Gene Expression and Prognostic Significance Using DNA Microarray

**Figure 6:**
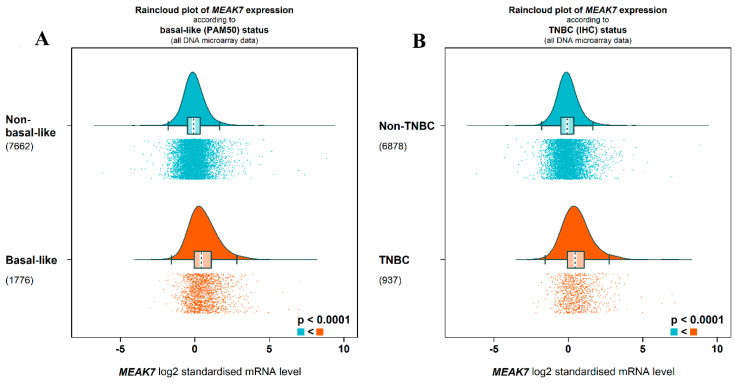
Distribution of MEAK7 expression according to molecular subtypes and TNBC status. (**A**) Raincloud plots showing MEAK7 mRNA expression in basal-like and non-basal-like breast cancer samples according to the PAM50 classification. (**B**) Distribution of MEAK7 mRNA expression levels in triple-negative breast cancer (TNBC) and non-TNBC cases, based on immunohistochemical (IHC) identification. The graphs present density curves (**top**), box plots (**middle**), and individual sample distributions (**bottom**) together. All analyses are based on DNA microarray data, and expression values are expressed as log2-standardized mRNA levels. In group comparisons, MEAK7 expression was found to be significantly higher in the basal-like and TNBC groups, with statistical significance calculated as *p* < 0.0001 for both analyses. Numbers in parentheses indicate the number of samples in each group.

In the bc-GenExMiner analysis (standardized log2 microarray scale, PAM50 classification), MEAK7 showed higher expression in basal-like tumors than in non-basal-like tumors (mean difference = 0.623; median difference = 0.524; *p* < 0.0001). Because the values are standardized, these differences represent an effect size on a normalized scale rather than a classical log2 fold change derived from raw expression ratios (Figure 6A). Standardized microarray analysis demonstrated higher MEAK7 expression in TNBC compared with non-TNBC tumors (mean difference = 0.63 and median difference = 0.55 on the standardized log2 scale; *p* < 0.0001), indicating a moderate effect size (Figure 6B).

**Figure 7:**
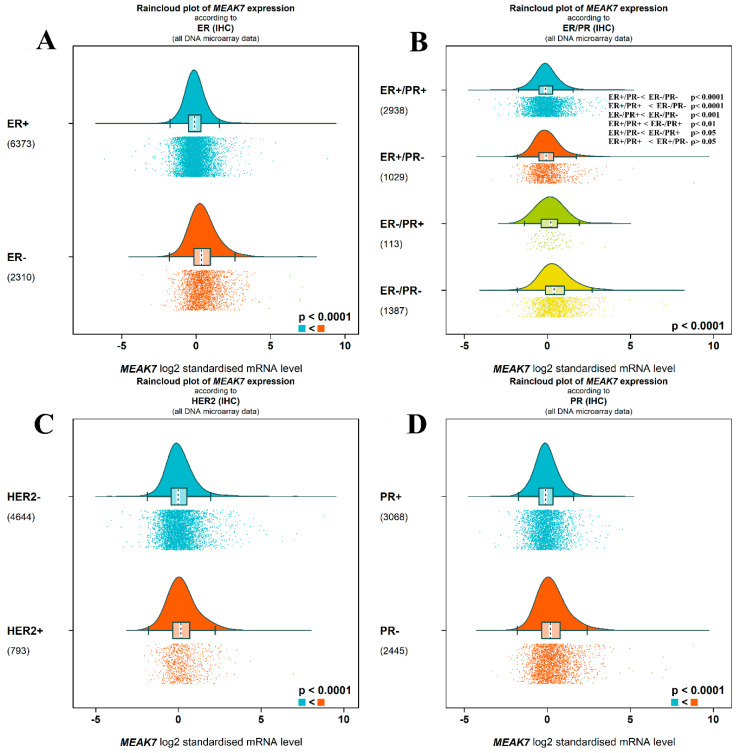
Distribution of MEAK7 expressions according to ER, PR, HER2, and ER/PR subgroups. The distribution of MEAK7 log2 standardized mRNA expression levels based on immunohistochemical (IHC) markers is shown using raincloud plots. (**A**) Comparison of ER positive (ER+) and ER negative (ER−) cases. (**B**) Distribution of MEAK7 expression in subgroups formed according to combinations of ER and PR (ER+/PR+, ER+/PR−, ER−/PR+, ER−/PR−). (**C**) Comparison of HER2 negative (HER2−) and HER2 positive (HER2+) cases. (**D**) Distribution of MEAK7 expression in PR positive (PR+) and PR negative (PR−) groups. All analyses are based on DNA microarray data. The graphs present density curves, box plots, and individual sample distributions together. Expression values are represented as log2 standardized mRNA levels. In group comparisons, the level of statistical significance was calculated as *p* < 0.0001. The numbers in parentheses indicate the sample size in each group.

In the analysis according to ER status (mean difference = 0.54), MEAK7 expression levels in ER-negative (n = 2310) cases were found to be significantly higher compared to the ER-positive (n = 6373) group (*p* < 0.0001) (Figure 7A). MEAK7 log2 standardized mRNA expression levels showed significant differences among the subgroups formed based on combinations of ER and PR (*p* < 0.0001). The ER+/PR+ group (n = 2938) had the lowest MEAK7 expression levels, while the ER−/PR− group (n = 1387) showed the highest expression values. In multiple comparisons, the ER+/PR+ group exhibited significantly lower MEAK7 expression compared to both the ER−/PR+ and ER−/PR− groups (mean difference = 0.24, *p* < 0.01; mean differences = 0.59, *p* < 0.0001, respectively). The ER+/PR− group (n = 1029) did not show statistically MEAK7 expression than the ER+/PR+ group (*p* > 0.05) but remained at lower levels compared to the ER−/PR− group (mean differences = 0.53, *p* < 0.001). The difference between the ER+/PR− and ER−/PR+ groups was not statistically significant (mean differences = 0.18, *p* > 0.05). The ER−/PR+ group (n = 113) exhibited lower MEAK7 expression compared to the ER−/PR− group (mean differences = 0.33, *p* < 0.001). Overall, MEAK7 expression appears to increase as hormone receptor negativity increases, reaching the highest values particularly in the double-negative (ER−/PR−) group (Figure 7B). In the analysis based on HER2 status, MEAK7 expression was found to be significantly higher in HER2-positive (n = 793) cases compared to the HER2-negative (n = 4644) group (mean difference = 0.17, *p* < 0.0001) (Figure 7C). Similarly, in the PR-negative group (n = 2445), MEAK7 expression levels were found to be significantly higher than in PR-positive cases (n = 3068) (mean difference = 0.36, *p* < 0.0001) (Figure 7D).

### 3.7. Survival Analyses of MEAK7 Expression in TNBC and Basal-like Breast Cancer

**Figure 8:**
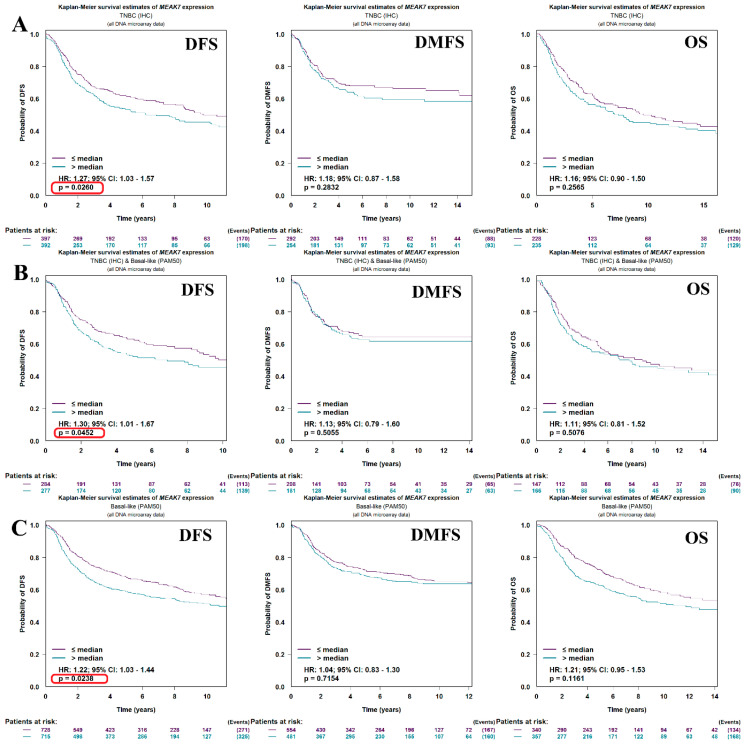
Survival analyses of MEAK7 expression in TNBC and basal-like breast cancer. Disease-free survival (DFS), distant metastasis-free survival (DMFS), and overall survival (OS) outcomes were analyzed using the Kaplan–Meier method based on MEAK7 expression levels (using the median as a cutoff point). All analyses are based on DNA microarray data. (**A**) DFS, DMFS, and OS analyses by MEAK7 expression in the TNBC (IHC) subgroup. (**B**) DFS, DMFS, and OS analyses by MEAK7 expression in cases that are both TNBC and exhibit basal-like (PAM50) characteristics. (**C**) DFS, DMFS, and OS analyses by MEAK7 expression in the basal-like (PAM50) breast cancer group. In the graphs, the purple line represents cases with MEAK7 expression below the median (≤median), and the turquoise line represents cases with MEAK7 expression above the median (>median). Hazard ratios (HRs), 95% confidence intervals (CIs), and *p*-values were calculated using the log-rank test. The lower panels show the number of patients at risk.

In TNBC cases, patients with MEAK7 expression above the median had significantly shorter DFS (HR = 1.27, 95% CI: 1.03–1.57, *p* = 0.0260). In contrast, no statistically significant difference was observed between the groups in terms of Distant metastasis-free survival (DMFS) (HR = 1.18, 95% CI: 0.87–1.58, *p* = 0.2832) and OS (HR = 1.16, 95% CI: 0.90–1.50, *p* = 0.2565) (Figure 8A). Among patients with TNBC and basal-like features, high MEAK7 expression was found to be significantly associated with shorter DFS (HR = 1.30, 95% CI: 1.01–1.67, *p* = 0.0452). However, in the same patient group, no significant difference associated with MEAK7 expression was observed regarding DMFS (HR = 1.13, 95% CI: 0.79–1.60, *p* = 0.5055) and OS (HR = 1.11, 95% CI: 0.81–1.52, *p* = 0.5076) (Figure 8B). In the basal-like breast cancer group, patients with MEAK7 expression above the median showed significantly worse DFS (HR = 1.22, 95% CI: 1.03–1.44, *p* = 0.0238). However, no statistically significant difference was found in the analyses for DMFS (HR = 1.04, 95% CI: 0.83–1.30, *p* = 0.7154) and OS (HR = 1.21, 95% CI: 0.95–1.53, *p* = 0.1161) (Figure 8C). Considering that multiple survival endpoints and subgroup analyses were assessed, the findings should be interpreted with appropriate caution in the context of multiple statistical testing.

### 3.8. In Silico CRISPR-Cas9 Targetability Analysis of MEAK7

A total of 20 gRNA oligos were designed for the CRISPR/Cas9-mediated knockout of the MEAK7 gene. Most of these guide RNAs exhibited high potency scores, ranging approximately from 81.0 to 99.3. For gRNAs with the highest activity scores (≥95), no off-target effects were observed, indicating high specificity for experimental applications. The top-ranked gRNA sequences are strong candidates for MEAK7 gene silencing, showing both high predicted cleavage efficiency and no off-target effects. However, some high-scoring gRNAs had potential off-target sites, requiring careful selection before validation. A G nucleotide should be preferred at the 5′ end of the gRNA sequence for U6 promoter transcription, with an additional G added if needed for lentiviral or plasmid-based expression systems. The findings reveal multiple CRISPR targeting sequences for MEAK7 with both high specificity and activity potential (Table 1).

### 3.9. Identification of Potential miRNA-Mediated Regulatory Mechanisms Targeting MEAK7

**Figure 9:**
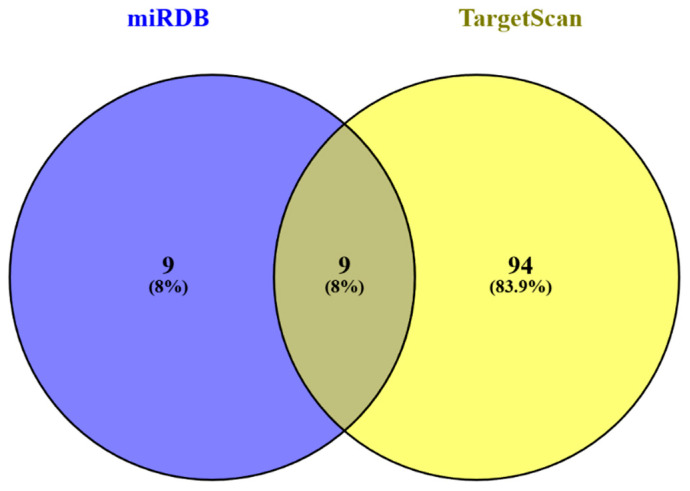
The Venn diagram presented herein delineates the overlap in predicted microRNAs (miRNAs) targeting MEAK7, as identified by the miRDB and TargetScan databases. Notably, nine miRNAs were consistently predicted by both databases. In contrast, miRDB uniquely identified nine miRNAs, while TargetScan uniquely predicted 94 miRNAs. The miRNAs common to both databases include hsa-miR-582-5p, hsa-miR-135b-5p, hsa-miR-135a-5p, hsa-miR-202-3p, hsa-miR-432-5p, hsa-miR-6855-3p, hsa-miR-4513, hsa-miR-3670, and hsa-miR-149-3p.

MicroRNA (miRNA) target prediction analysis for MEAK7 was performed using the miRDB and TargetScan databases. A comparative analysis revealed that nine miRNAs were consistently predicted by both platforms. Additionally, nine miRNAs were uniquely identified by miRDB, whereas 94 miRNAs were exclusively predicted by TargetScan. The miRNAs predicted by both databases included hsa-miR-582-5p, hsa-miR-135b-5p, hsa-miR-135a-5p, hsa-miR-202-3p, hsa-miR-432-5p, hsa-miR-6855-3p, hsa-miR-4513, hsa-miR-3670, and hsa-miR-149-3p. These shared miRNAs were selected for further analyses (Figure 9 and Table 2).

### 3.10. LncRNAs Associated with MEAK7 and Triple-Negative Breast Cancer

**Figure 10:**
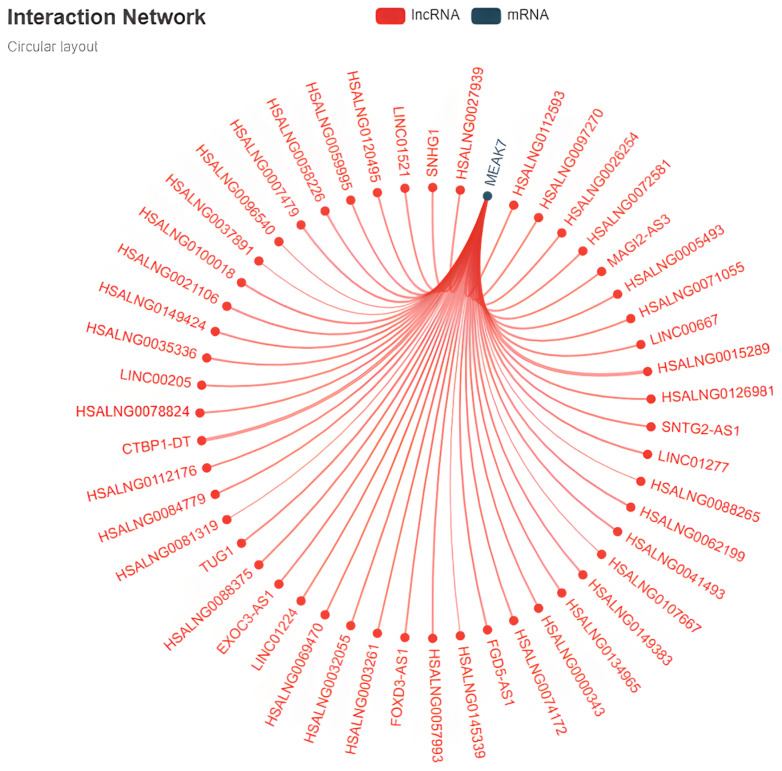
Visualization of MEAK7 and TNBC-associated lncRNA networks. The lncRNA–mRNA co-expression interaction network for MEAK7 was derived using the LncNet tool within the LncExpDB Version 2.0 database. This network presents lncRNAs associated with MEAK7 in a circular layout, based on Spearman correlation analysis.

The lncRNA–mRNA co-expression interaction network, constructed utilizing the LncNet tool available in the LncExpDB Version 2.0 database, is presented. Through Spearman correlation-based analysis, numerous lncRNAs exhibiting significant associations with MEAK7 were identified, with the majority demonstrating a positive co-expression pattern (Figure 10). lncRNAs associated with TNBC were compiled using the LncRNADisease database. This analysis revealed that lncRNAs such as MIR31HG, LINC-ROR, HIF1A-AS2, MALAT1, LINC00993, AK124454, RP11-434D9.1, LINC00052, BC016831, and IGKV, which have been linked to TNBC, may suggest potential regulatory interactions surrounding MEAK7 (Table 3). Notably, the presence of lncRNAs related to proliferation, invasion, metastasis, and chemotherapy resistance implies that MEAK7 may be associated with an aggressive tumor phenotype and treatment response.

### 3.11. Analysis of Correlative Associations Between MEAK7 (TLDC1) and Selected miRNAs in the BRCA Cohort

**Figure 11:**
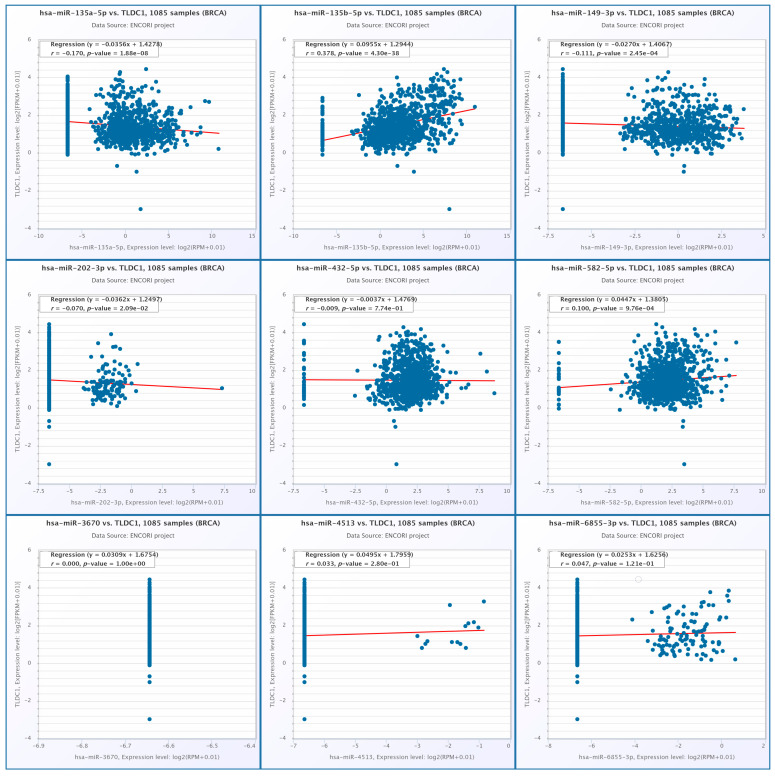
The correlation analysis between MEAK7 expression and selected miRNAs in breast cancer (ENCORI, n = 1085) is presented. In each panel, individual patient samples are represented as dots, with a red line indicating the linear regression. The correlation coefficient (r) and *p*-value are provided in each graph. Expression levels are depicted using a log2(RPM + 0.01) transformation.

Correlation analyses were performed to examine the relationship between MEAK7 expression and selected miRNAs within the BRCA cohort (n = 1085) using the ENCORI database. The results indicated a weak negative correlation between hsa-miR-135a-5p and MEAK7 (TLDC1) (r = −0.170, *p* = 1.88 × 10^−8^). Conversely, a moderate positive correlation was identified between hsa-miR-135b-5p and MEAK7 (r = 0.378, *p* = 4.30 × 10^−38^). Additionally, weak negative correlations were observed for hsa-miR-149-3p (r = −0.111, *p* = 2.45 × 10^−4^) and hsa-miR-202-3p (r = −0.070, *p* = 2.09 × 10^−2^). No significant correlation was detected for hsa-miR-432-5p (r = −0.009, *p* = 0.774). A weak yet statistically significant positive correlation was found between hsa-miR-582-5p and MEAK7 (r = 0.100, *p* = 9.76 × 10^−4^). No significant correlations were observed for hsa-miR-3670 (r = 0.000, *p* = 1.00), hsa-miR-6855-3p (r = 0.047, *p* = 0.121), and hsa-miR-4513 (r = 0.033, *p* = 0.280) (Figure 11).

### 3.12. Analysis of Correlational Associations Between Selected lncRNAs and MEAK7 in the BRCA Cohort

**Figure 12:**
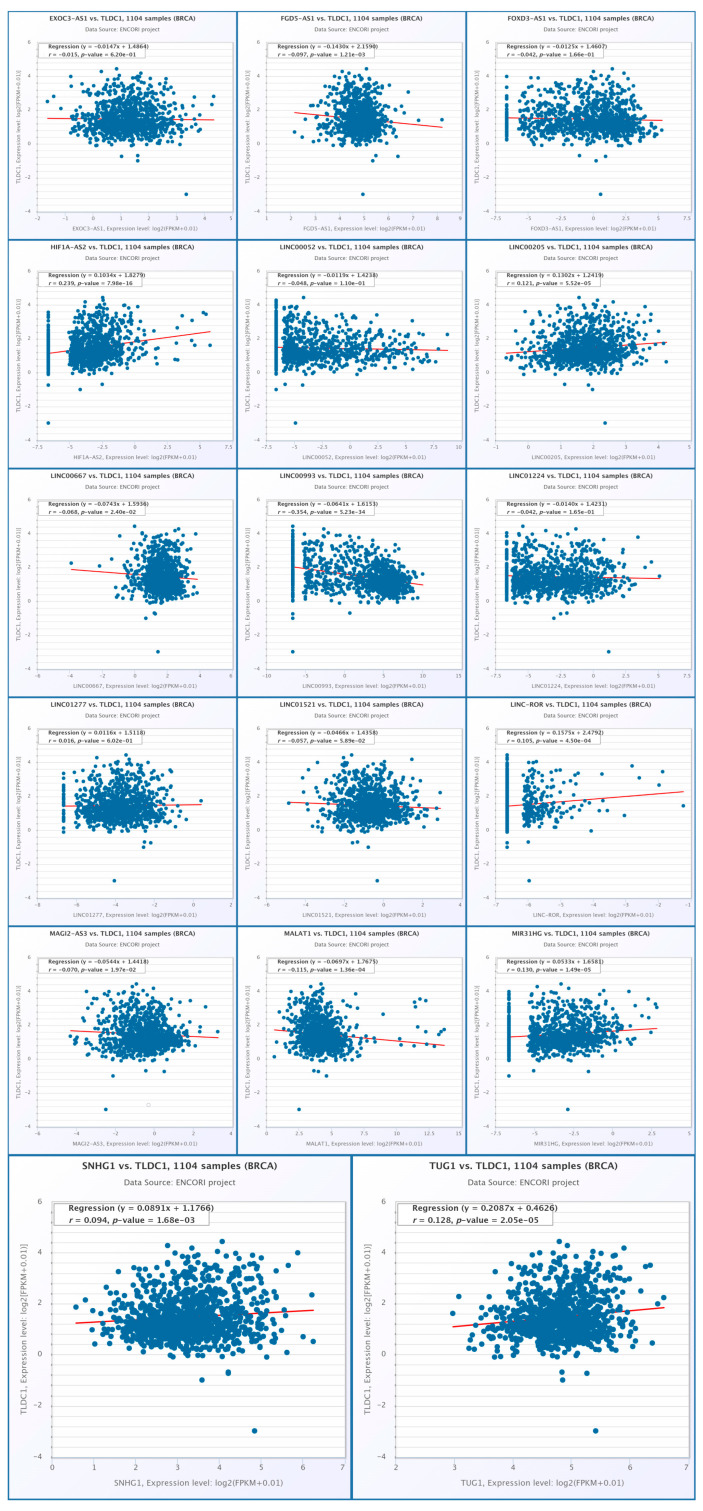
In the analysis of the correlation between MEAK7 expression and selected long non-coding RNAs (lncRNAs) in breast cancer, utilizing the ENCORI dataset (n = 1104), each panel illustrates the distribution of MEAK7 expression levels alongside the corresponding lncRNA, accompanied by a linear regression curve. Individual patient samples are represented as dots, with a red line indicating the linear regression. Each graph displays the correlation coefficient (r) and the *p*-value. Expression levels are presented using a log2(RPM + 0.01) transformation.

The correlation between MEAK7 expression and selected lncRNAs in the BRCA cohort (n = 1104) was assessed utilizing the ENCORI database. The analysis identified several lncRNAs with a positive correlation, including HIF1A-AS2 (r = 0.239, *p* = 7.98 × 10^−16^), LINC00205 (r = 0.121, *p* = 5.52 × 10^−5^), LINC-ROR (r = 0.105, *p* = 4.50 × 10^−4^), MIR31HG (r = 0.130, *p* = 1.49 × 10^−5^), SNHG1 (r = 0.094, *p* = 1.68 × 10^−3^), and TUG1 (r = 0.128, *p* = 2.05 × 10^−5^). Conversely, lncRNAs exhibiting a negative correlation included LINC00993 (r = −0.354, *p* = 5.32 × 10^−34^), FGD5-AS1 (r = −0.097, *p* = 1.21 × 10^−3^), LINC00667 (r = −0.068, *p* = 2.40 × 10^−2^), MAGI2-AS3 (r = −0.070, *p* = 1.97 × 10^−2^), and MALAT1 (r = −0.115, *p* = 1.36 × 10^−4^). Other lncRNAs, such as EXOC3-AS1, FOXD3-AS1, LINC00052, LINC01224, LINC01277, and LINC01521, demonstrated low correlation coefficients, with no statistically significant associations observed (all *p* > 0.05) (Figure 12).

## 4. Discussion

This study is one of the first comprehensive and multi-layered bioinformatic analyses conducted on the expression profile, epigenetic regulation, therapeutic potential and prognostic significance of the MEAK7 gene in BC and BC subtypes. The particular focus on TNBC is characterized by an aggressive clinical course, limited treatment options, and poor prognosis further enhances the study’s originality in the literature and its clinical significance.

Our analyses have revealed that MEAK7 expression is significantly increased in BC tissues compared to normal breast tissue, and this increase is especially pronounced in TNBC and basal-like (PAM50). This observation implies a possible correlation between MEAK7 and the development of more aggressive molecular tumor phenotypes. This is consistent with prior research indicating that TNBC and basal-like breast cancers are characterized by increased activation of growth- and metabolism-related signaling pathways, particularly the PI3K/AKT/mTOR axis [40,41]. The progressive increase in MEAK7 expression correlates with the rise in hormone receptor negativity (ER−/PR−), thereby reinforcing the association of MEAK7 with hormone-independent tumor behavior. This characteristic is a well-documented hallmark TNBC biology and is closely associated with poor prognosis and therapeutic resistance [2,42]. While MEAK7 expression was statistically elevated in the TNBC group, the moderate standardized mean difference between the TNBC and non-TNBC groups suggests that MEAK7 demonstrated a discernible yet not substantial effect size.

Survival analyses have shown that high MEAK7 expression is significantly associated with poor DFS, particularly in TNBC and basal-like. This condition aligns with the well-documented clinical characteristics of TNBC, which is characterized by a heightened risk of early recurrence and rapid disease progression, particularly within the initial years following diagnosis [43,44]. Therefore, a previous study has reported an association between upregulated MEAK7 expression and lymph node metastasis in BC [17]. Given that DFS encompasses local and regional disease recurrence, including lymph node involvement, our finding that elevated MEAK7 expression is associated with shorter DFS is biologically consistent with these earlier observations. While MEAK7 expression exhibited significant correlations with OS and RFS, the lack of significance in PPS and TNBC-specific OS, alongside the selective significance observed for TNBC DFS, suggests that the prognostic impact of MEAK7 is unlikely to be consistent across all survival endpoints. The current analysis may suggest clinical relevance that is both context-dependent and specific to certain subtypes. Such variability may stem from intrinsic biological heterogeneity among breast cancer subtypes, differences in the clinical interpretation and timing of survival endpoints, and potential statistical inflation due to multiple hypothesis testing in large-scale public datasets. Consequently, MEAK7 should be regarded as a provisional and context-sensitive prognostic marker rather than a universally applicable biomarker. Prospective validation and mechanistic studies are thus necessary to ascertain its precise clinical utility across diverse molecular and therapeutic contexts. Therefore, this pattern aligns with previous findings, which suggest that TNBC relapse frequently occurs early and locally before distant dissemination becomes clinically apparent [45,46,47]. The prognostic impact observed for MEAK7 in this study falls within the range reported for several previously described biomarkers in TNBC. Established prognostic markers in TNBC, including proliferation-related markers such as Ki-67, immune-related signatures such as PD-L1 expression and tumor-infiltrating lymphocytes (TILs), and molecular regulators associated with PI3K/mTOR signaling pathways, have demonstrated comparable hazard ratios in survival analyses [48]. In this context, the prognostic magnitude observed for MEAK7 suggests that it may represent a biologically relevant marker associated with aggressive tumor behavior. However, additional studies integrating MEAK7 expression with established clinical and molecular markers will be required to determine its incremental prognostic value in TNBC. Interestingly, the PPS analysis showed a hazard ratio direction opposite to that observed for OS and RFS. Although this association was not statistically significant, such discrepancies may arise from differences in post-progression treatment strategies, patient heterogeneity, and the limited statistical power of subgroup analyses. The clinical significance of MEAK7 can be more comprehensively understood when considered alongside established TNBC biomarkers such as Ki-67, EGFR, and PD-L1, which are evaluated at the protein level via immunohistochemistry. Our findings suggest that MEAK7 exhibits greater consistency at the transcript level, indicating that RNA-based assays may offer more reliable detection. This underscores MEAK7’s potential advantage as a biomarker that complements protein-based markers, particularly in instances where transcriptional changes precede protein alterations. Therefore, MEAK7 may be more appropriately integrated into transcriptomic panels rather than protein-based diagnostic workflows.

Moreover, the identification of MEAK7 as an independent prognostic factor, even after controlling for age and tumor stage in a multivariate Cox regression analysis, underscores the clinical significance of this gene. Independent prognostic biomarkers are particularly crucial in the context of TNBC, where conventional clinicopathological parameters frequently fall short in adequately assessing tumor aggressiveness and the risk of relapse [6,49,50].

Epigenetic analyses have demonstrated that the MEAK7 promoter region exhibits relative hypomethylation in the TNBC, which is considered a potential regulatory mechanism contributing to the elevated MEAK7 expression observed in TNBC. Promoter hypomethylation is a well-documented epigenetic characteristic associated with transcriptional activation in cancer and is frequently reported in TNBC, where epigenetic dysregulation plays a pivotal role in promoting oncogene expression and tumor aggressiveness [3,51]. Considering the combined analysis of transcriptomic and methylation data in this study, it appears that the upregulation of MEAK7 expression is not merely coincidental. Instead, it likely represents a biologically regulated process aligned with established epigenetic mechanisms active in TNBC.

One of the most robust aspects of this study is the comprehensive evaluation of MEAK7, not only within the general BC cohort but also specifically within the clinically aggressive TNBC. The literature on the role of MEAK7 in solid tumors is notably sparse, and existing data does not offer an evaluation specific to TNBC. In this context, the findings presented herein establish a substantial foundation for considering MEAK7 as a potential prognostic biomarker in TNBC biology and as a molecular target for future therapeutic interventions.

The analysis of gRNA design indicates that experimental CRISPR strategies targeting the MEAK7 gene are functionally viable. The identification of gRNAs with high potency scores and no off-target effects facilitates the direct investigation of MEAK7’s cellular functions through genetic silence. Given that MEAK7 is a protein associated with mTORC1, the knockout of this gene could contribute to the mechanistic elucidation of oncogenic processes such as cellular proliferation, metabolic reprogramming, and tumor cell survival, which are significant for the TNBC. In this context, the findings provide a crucial link that could advance bioinformatic prognostic analyses in BC and other solid tumors to the experimental stage. In addition, CRISPR gRNA design analysis was included as a preliminary translational exploration to evaluate the potential feasibility of MEAK7-targeted genome editing strategies. This analysis was not intended as functional validation but rather as an initial step to support future experimental studies investigating the therapeutic targetability of MEAK7.

In the context of TNBC, it is established that the microRNAs miR-149-3p and miR-135a-5p, which have been functionally investigated, are implicated in competing endogenous RNA (ceRNA) and long non-coding RNA (lncRNA)-mediated networks, as well as in metastatic phenotypes. Their downregulation is associated with cancer progression and unfavorable prognosis [51,52]. Considering the inverse correlation observed between these miRNAs and MEAK7 within our BRCA cohort, it is posited that MEAK7 may play a role in cancer pathogenesis through its involvement in miRNA and ceRNA/lncRNA-mediated regulatory pathways. The observed patterns could offer a novel molecular framework for MEAK7-focused research. Conversely, the positive association identified between MEAK7 expression and miR-135b-5p in the BRCA cohort further substantiates the hypothesis that MEAK7 may hold a significant position within miRNA-mediated regulatory networks. Indeed, Research has documented that miR-135b-5p is upregulated in TNBC and facilitates oncogenic processes via the circ_0000977/miR-135b-5p axis by inhibiting tumor suppressor genes such as APC and GATA3 [53,54]. The findings of this study indicate that the MEAK7-associated miRNA patterns may be connected to RNA-based regulatory mechanisms that are specific to TNBC. Regarding lncRNAs, elevated expression of HIF1A-AS2 has been correlated with lymph node metastasis, distant metastasis, poor histological grade, and reduced overall survival in TNBC. Furthermore, HIF1A-AS2 has been identified as an independent prognostic marker associated with poor outcomes [55]. In our study, the observed positive correlation between MEAK7 and HIF1A-AS2 indicates that MEAK7 may be involved in molecular networks linked to aggressive tumor behavior and unfavorable clinical outcomes in TNBC. The literature demonstrates that LINC00993 is significantly downregulated in TNBC; however, its elevated expression correlates with improved survival and exerts a tumor-suppressive effect by arresting the cell cycle at the G0/G1 phase. Additionally, it has been documented that LINC00993 inhibits proliferation and tumor growth both in vitro and in vivo by upregulating cell cycle regulators such as p16INK4A, p14ARF, p21, and p53 [56]. In this context, the observed negative correlation between MEAK7 and LINC00993 within our BRCA cohort indicates that MEAK7 may be inversely related to tumor-suppressive pathways in TNBC, potentially exerting functionally opposing biological effects on cell cycle and proliferation processes. Consequently, the MEAK7–LINC00993 axis may be regarded as a potential regulatory network component that reflects the equilibrium between proliferative and suppressive signals in the pathogenesis of TNBC.

Interestingly, although transcriptomic analyses demonstrated a significant upregulation of MEAK7 mRNA in BC tissues, the immunohistochemical staining obtained from the HPA showed relatively low-to-moderate protein expression levels. This apparent discordance between mRNA and protein abundance has been reported for many genes and may reflect multiple regulatory mechanisms operating at the post-transcriptional or translational level [57]. For instance, miRNA-mediated translational repression, differences in protein stability or degradation, and post-transcriptional regulatory mechanisms may limit the translation of MEAK7 transcripts into detectable protein levels [58]. In addition, tissue-specific regulatory mechanisms and technical factors related to antibody sensitivity or epitope accessibility in immunohistochemistry datasets may also contribute to this observation [59]. From a translational perspective, these findings may highlight that MEAK7-driven tumorigenic processes may be regulated at the RNA level, and its clinical utility may be better reflected in gene expression-based assays rather than protein-level detection. Notably, this discrepancy may suggest that MEAK7 may be more suitable as a transcript-level biomarker rather than a protein-based marker, which should be considered when interpreting its clinical applicability Therefore, further experimental studies integrating transcriptomic and proteomic analyses will be required to clarify the biological regulation of MEAK7 expression in BC.

Our integrative bioinformatics analyses consistently reveal associations between MEAK7 expression and adverse clinical outcomes, particularly in TNBC and basal-like. However, these findings are predominantly based on transcriptomic correlations derived from publicly accessible datasets. Consequently, the present study does not conclusively establish MEAK7 as an oncogenic driver but rather posits it as a potential biomarker linked to aggressive tumor biology. MEAK7 has been implicated in the regulation of the mTOR signaling network, a pathway known to facilitate tumor growth, metabolic reprogramming, and therapeutic resistance across various cancers [60]. Nonetheless, the precise mechanistic role of MEAK7 in tumor progression, particularly in BC, and its causal contribution to oncogenic processes remain to be elucidated. Therefore, future experimental investigations—including functional assays such as gene silencing or overexpression models and pathway analyses—are imperative to ascertain whether MEAK7 constitutes a biologically actionable target or merely a molecular correlate of aggressive disease phenotypes. It is important to acknowledge that some datasets employed in this study are partially sourced from TCGA-based cohorts. Consequently, certain results may be affected by overlapping patient populations and should be interpreted with due caution.

### Limitation

The study has limitations. As analyses used publicly accessible transcriptomic and microarray datasets, findings require independent experimental validation. The potential overlap of datasets across multiple bioinformatic platforms, particularly those derived from the TCGA cohort. Such partial cohort overlaps may influence statistical significance and should therefore be considered when interpreting the results. These results should be interpreted cautiously as hypothesis-generating rather than conclusive evidence. Moreover, the lack of formal multiple testing correction represents a limitation and may increase the risk of false-positive findings. A key consideration is the distinction between statistical significance and effect size. While increased MEAK7 expression was linked to adverse survival outcomes across datasets, the hazard ratios suggest a modest prognostic effect. In large-scale transcriptomic cohorts, small differences may achieve statistical significance due to large sample sizes rather than substantial clinical impact. Therefore, MEAK7’s prognostic significance should be interpreted with emphasis on effect estimates and confidence intervals, not just *p*-values. Future studies with multivariable models, discrimination metrics, and prospective validation cohorts are needed to determine MEAK7’s clinical utility as a biomarker. Nevertheless, a significant limitation is that only in silico gRNA design has been conducted. To validate actual cleavage efficiency, it is necessary to employ T7E1 or Surveyor assays, Sanger/NGS-based indel analysis, and confirm MEAK7 expression through Western blot or qPCR. It should also be noted that the HER2-positive in the UALCAN analysis included a relatively small number of samples (n = 17), which may limit the statistical power and reliability of subgroup-specific inferences. Therefore, these findings should be interpreted with caution. Another limitation is that the multivariate Cox analyses were performed using the DoSurvive platform, where the available covariates are limited. Therefore, important prognostic variables such as tumor grade, nodal status, treatment modality, molecular subtype, and mutational burden could not be included in the model. Furthermore, a comprehensive genome-wide assessment of gRNAs with potential off-target effects is essential for ensuring translational safety. The discrepancy between MEAK7 mRNA and protein expression levels represents a limitation, as it suggests that its biomarker potential may be more accurately reflected at the transcript level rather than at the protein level. Therefore, the findings of this study should be interpreted as hypothesis-generating observations that may provide a conceptual framework for future experimental and clinical investigations rather than definitive evidence of causal relationships.

## 5. Conclusions

This study is the first systematic and multi-platform analysis to reveal that MEAK7 is associated with high expression and adverse clinical outcomes, particularly in aggressive BC subtypes such as TNBC, its relationship with miRNAs and lncRNAs, and its therapeutically targetable nodes. When all these results are evaluated, MEAK7 may be a promising transcript-level prognostic biomarker. However, further studies are needed to explore the potential of MEAK7 as a novel prognostic biomarker and therapeutic target.

## Figures and Tables

**Table 1:** CRISPR/Cas9 gRNAs targeting MEAK7.

gRNA Sequence	Potency Score	Off-Target Status	Gene ID	Transcript ID	Gene Symbol	Gene Description
**CCTGTTGAAAGGAAACTCCG**	99.3	No off-target identified	57707	NM_020947	MEAK7	MTOR associated protein, eak-7 homolog
**CAGTCATAGTCCAGCCACTG**	95.8	No off-target identified	57707	NM_020947	MEAK7	MTOR associated protein, eak-7 homolog
**CGTTGATGTACATGACAGAG**	94.6	No off-target identified	57707	NM_020947	MEAK7	MTOR associated protein, eak-7 homolog
**CAGCCTGGTGACCATCTCTG**	93.2	No off-target identified	57707	NM_020947	MEAK7	MTOR associated protein, eak-7 homolog
**GAGGGTCGACCTGACAGGGA**	91.6	No off-target identified	57707	NM_020947	MEAK7	MTOR associated protein, eak-7 homolog
**ATGGTCACCAGGCTGTATGA**	91.3	No off-target identified	57707	NM_020947	MEAK7	MTOR associated protein, eak-7 homolog
**ACATGACAGAGAGGACATCC**	88.2	No off-target identified	57707	NM_020947	MEAK7	MTOR associated protein, eak-7 homolog
**GCTGAGAGGCTGGACTGGGA**	86.3	No off-target identified	57707	NM_020947	MEAK7	MTOR associated protein, eak-7 homolog
**CCAGGCTGTATGATGGCATG**	86.0	No off-target identified	57707	NM_020947	MEAK7	MTOR associated protein, eak-7 homolog
**GTTTGCCTCTTGCTCTTGGG**	83.7	No off-target identified	57707	NM_020947	MEAK7	MTOR associated protein, eak-7 homolog
**CATGACAAGCATGTGTTCGG**	82.4	No off-target identified	57707	NM_020947	MEAK7	MTOR associated protein, eak-7 homolog
**AGCCACAGACAGGAGCTGAG**	99.0	Potential off-targets identified	57707	NM_020947	MEAK7	MTOR associated protein, eak-7 homolog
**TGTGGCCACATCACTCACCG**	99.0	Potential off-targets identified	57707	NM_020947	MEAK7	MTOR associated protein, eak-7 homolog
**GGAGCTCAGACGAAAAGAGC**	93.1	Potential off-targets identified	57707	NM_020947	MEAK7	MTOR associated protein, eak-7 homolog
**TGGACTGGGAAGGAAGCCCC**	92.1	Potential off-targets identified	57707	NM_020947	MEAK7	MTOR associated protein, eak-7 homolog
**ACGAAAAGAGCAGGCACCAG**	86.3	Potential off-targets identified	57707	NM_020947	MEAK7	MTOR associated protein, eak-7 homolog
**TCAGATCAAGAGACGAGCAC**	83.3	Potential off-targets identified	57707	NM_020947	MEAK7	MTOR associated protein, eak-7 homolog
**TGTGGCCATATTCCTGAGTG**	83.3	Potential off-targets identified	57707	NM_020947	MEAK7	MTOR associated protein, eak-7 homolog
**AGGGTCCCCGGTGAGTGATG**	81.0	Potential off-targets identified	57707	NM_020947	MEAK7	MTOR associated protein, eak-7 homolog
**GGAAGCTCTTCCCCCAGAGA**	81.0	Potential off-targets identified	57707	NM_020947	MEAK7	MTOR associated protein, eak-7 homolog

Note: (1) Potency Score is calculated based on computational prediction of the knockout potency of the gRNA, with a higher score representing higher predicted potency. (2) Off-target status is determined by checking against all known gene sequences in the genome. (3) U6 promoter prefers a starting “G” for transcription. If you use U6 promoter and the 20mer oligo does not start with a G, it is recommended to add an extra G to the 5′-end of the 20mer oligo (making it a 21mer oligo). All gRNAs target the MEAK7 gene (Gene ID: 57707; Transcript ID: NM_020947).

**Table 2:** The miRNAs are associated with *MEAK7* with combination of TargetScanHuman8.0 and miRDB databases.

Predicted miRNAs for MEAK7	
**miRDB databases** **TargetScanHuman8.0**	hsa-miR-582-5p, hsa-miR-135b-5p, hsa-miR-135a-5p, hsa-miR-202-3p, hsa-miR-432-5p, hsa-miR-6855-3p, hsa-miR-4513, hsa-miR-3670, hsa-miR-149-3p

**Table 3:** lncRNAs associated with triple-negative breast cancer (TNBC) was obtained from the LncRNADisease database.

LncRNA Name	Disease Name	Dysfunction Type	Description	Reference
MIR31HG	breast cancer	Epigenetics	miR-31 and its host gene lncRNA LOC554202 (MIR31HG) are regulated by promoter hypermethylation in triple-negative breast cancer. Both miR-31 and the host gene LOC554202 are down-regulated in the TNBC cell lines of basal subtype and over-expressed in the luminal counterparts.	[34]
LINC-ROR	breast cancer	Expression	LincRNA-RoR is upregulated in TNBC and in metastatic disease and knockdown restores miR-145 expression. The lincRNA-RoR/miR-145/ARF6 pathway is critical to TNBC metastasis and could serve as biomarkers or therapeutic targets for improving survival.	[35]
HIF1A-AS2	breast cancer	Regulation	HIF1A-AS2 and AK124454 promoted cell proliferation and invasion in TNBC cells and contributed there to paclitaxel resistance.	[36]
AK124454	breast cancer	Regulation	HIF1A-AS2 and AK124454 promoted cell proliferation and invasion in TNBC cells and contributed there to paclitaxel resistance.	[36]
RP11-434D9.1	breast cancer	N/A	It is correlated with TNBC occurrence	[37]
LINC00052	breast cancer	N/A	It is correlated with TNBC occurrence	[37]
BC016831	breast cancer	N/A	It is correlated with TNBC occurrence	[37]
IGKV	breast cancer	N/A	It is correlated with TNBC occurrence	[37]
MALAT1	breast cancer	Expression	MALAT1 may be a target for TNBC therapy.	[38]
LINC00993	breast cancer	Regulation	It may be involved in the development and/or progression of TNBC.	[39]

## Data Availability

The data used in this study were obtained from the public database TCGA and others.

## Abbreviations

The following abbreviations are used in this manuscript:

BC	Breast Cancer
TNBC	Triple-Negative Breast Cancer
MEAK7	mTORC1-associated protein
TCGA	The Cancer Genome Atlas
GEO	Gene Expression Omnibus
GEPIA3	Gene Expression Profiling Interactive Analysis 3
HPA	Human Protein Atlas
TPM	Transcripts per million
IHC	Immunohistochemistry
UALCAN	University of Alabama at Birmingham Cancer Data Analysis Portal
KMplot	Kaplan–Meier Plotter
OS	Overall survival
RFS	Relapse-free survival
PFS	Progression-free survival
GO	Gene Ontology
FDR	False discovery rate
lncRNA	Long non-coding RNA
miRNA	MicroRNA
